# Ferulic Acid in Animal Models of Alzheimer’s Disease: A Systematic Review of Preclinical Studies

**DOI:** 10.3390/cells10102653

**Published:** 2021-10-05

**Authors:** Er-Jin Wang, Ming-Yue Wu, Jia-Hong Lu

**Affiliations:** State Key Laboratory of Quality Research in Chinese Medicine, Institute of Chinese Medical Sciences, University of Macau, Macao, China; wangerjin.1619@connect.um.edu.mo (E.-J.W.); mingyuewu@umac.mo (M.-Y.W.)

**Keywords:** Alzheimer’s disease, ferulic acid, animal models, systematic review

## Abstract

Alzheimer’s disease (AD) is a neurodegenerative disease with a high incidence in the elderly. Many preclinical studies show that a natural product, ferulic acid (FA), displays neuroprotective effects in AD models. This review aims to systematically review and meta-analyze published pre-clinical researches about the effects, mechanism, and clinical prospects of FA in the treatment of AD. According to the pre-determined search strategy and inclusion criteria, a total of 344 animals in 12 papers were included in the meta-analysis. We used the fixed effects model to analyze data and I^2^ and *p* values to indicate heterogeneity. Results show that FA treatment can effectively improve rodents’ spatial memory ability in MWM and Y maze experiments (I^2^ ≥ 70, *p* < 0.005), and reduce the deposition of Aβ in the brains of various model animals (I^2^ ≥ 50, *p* < 0.005). The potential mechanisms include anti-amyloidogenesis, anti-inflammation, anti-oxidation, mitochondrial protection, and inhibition of apoptosis. In conclusion, we systematically review and meta-analyze the literature reporting the effects of FA treatment on AD rodent models and solidify the benefits of FA in reducing Aβ deposition and improving memory in preclinical experiments. We also point out the limitations in the current research design and provide a strategy for the production research of FA in the future.

## 1. Introduction

Alzheimer’s disease (AD) is an age-related progressive degenerative disease of the central nervous system with multiple causes. The onset of AD is closely related to age, genetic factors, and environmental factors. However, None of any existing theories can fully explain the complex pathology of AD at present. The clinical manifestations are memory and cognitive function impairment usually accompanied by mental behavior abnormality and personality change [1]. Its characteristic pathological feature is the deposition of abnormal protein aggregates, namely β amyloid protein (Aβ) in extraneuronal space (senile plaques) and hyperphosphorylated tau protein within neurons (neurofibrillary tangles).

AD is distributed worldwide and is the most common cause of dementia. With the gradual aging of the population in developed and developing countries, AD has become the fourth leading cause of human death and has a huge economic impact [2]. It is estimated that, by 2050, more than 1.315 billion people worldwide will have AD [3].

Ferulic acid (FA), 4-hydroxy-3-methoxycinnamic acid (C_10_H_10_O_4_, MW = 194.18), is a type of phenolic acid and a derivative of cinnamic acid. It is widely present in nature and distributed in a variety of fruits, vegetables, and grains, and also one of the active components in some Chinese medicinal herbs such as *Angelica acutiloba*, *Ligusticum striatum*, *Cimicifuga dahurica*, and *Crocus sativus* [4,5]. A variety of herbal medicines containing FA are believed to have the effects of promoting blood circulation, removing blood stasis, and reducing inflammation and pain in traditional Chinese medicine. Modern medical research has found that FA is a multi-functional compound with anti-inflammatory, antioxidant, anti-amyloid, neurotrophic, anti-microbial, antiallergic, hepatoprotective, anti-carcinogenic, and anti-thrombotic activities [6,7,8]. Recently, many studies have shown that FA is neuroprotective in a variety of neurodegenerative diseases models, both in vivo to *in vitro*, and especially in AD models.

In this article, we systematically review and meta-analyze the literature reporting the effects of FA treatment on AD rodent models by evaluating the quality of these articles and the benefits of FA in reducing Aβ deposition and improving memory in preclinical experiments. We also point out the weak points of current studies and suggest directions for productive future study of FA.

## 2. Methods

### 2.1. Search Strategy

We used “ferulic acid” and “Alzheimer’s disease” (or “Alzheimer Disease” or “AD”) as keywords to search in four databases: PubMed, EM-BASE, Web of Science, and CNKI. We designed search strategies according to the characteristics of the different databases. We searched up through 31 December 2020. The headings and abstracts of the selected articles were screened according to predetermined inclusion and exclusion criteria. This part of the work was performed independently by Er-jin Wang and Ming-yue Wu. Then, for articles for which the abstract did not provide necessary information, we obtained the full text and screened that. In case of ambiguity, selection decision was made after discussion with the third author.

### 2.2. Inclusion and Exclusion Criteria

Only those research studies that used rodents as AD model animals, and that included both a feurlic acid-treated group and a control group, were chosen. Articles that did not contain raw data or for which the full text could not be accessed were excluded. The specific inclusion and exclusion criteria are shown in Table 1.

### 2.3. Data Extraction

We extracted the following items from the included studies: basic information (article title, author, publication year), test animals (species, gender, quantity), AD models, intervention (mode, dose, duration), and experimental results (behavioral change, neuropathological change, biochemical change). When a study contained multiple outcomes, it was treated as independent data.

### 2.4. Methodological Quality of Studies

Referring to the quality evaluation criteria of animal experimental systems designed by Malcolm et al. [9] and Hooijmans et al. [10], the methods provided by Collaborative Approach to Meta-Analysis and Review of Animal Data from Experimental Stroke (CAMARADES), and taking into account the characteristics of AD diseases, we designed an “AD animal research quality evaluation form” to evaluate the methodological quality of each study. There were 10 categories of evaluation. When it could be clearly determined that the article met the criteria in one category, a score of one point was given. The maximum score possible was 10. The highest score for each study is 10.

### 2.5. Statistical Analysis

The mean and standard deviation (SD) of the required data were collected using Digit software. In order to make the results of the same indicator tested by different methods more comparable, we evaluated the standardized mean difference between experimental data. Review manager 5.3 software was used for meta-analysis. A fixed effects model was used to process the data. Q test and I^2^ statistics were used to judge heterogeneity. Heterogeneity was considered to exist when *p* < 0.05. I^2^ = 0% means no heterogeneity; 0 < I^2^ ≤ 25% means mild heterogeneity; 25% < I^2^ ≤ 75% means moderate heterogeneity; and I^2^ > 75% means a high degree of heterogeneity.

## 3. Results

This section may be divided by subheadings. It should provide a concise and precise description of the experimental results, their interpretation, as well as the experimental conclusions that can be drawn.

### 3.1. Literature Screening

**Figure 1:**
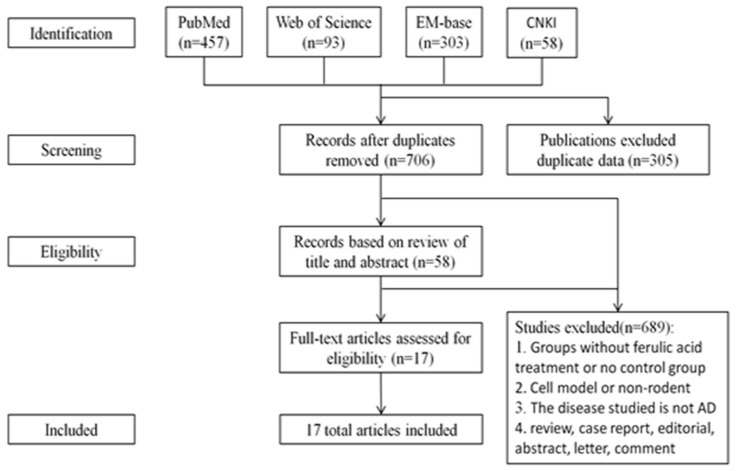
Research methodology for review process.

**Figure 2:**
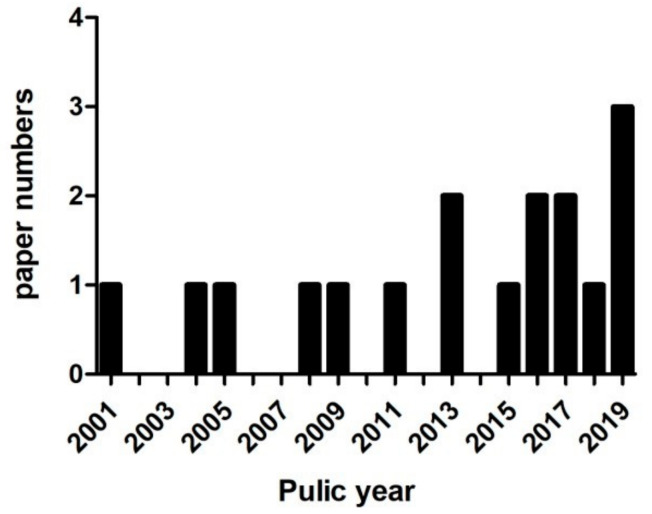
Publication trend per year.

Our pre-set search strategy returned 457, 193, 303, and 58 publications from PubMed, Web of Science, EM-base, and CNKI, respectively. After removing duplicates, 706 remained. After browsing the title and abstract or reading the full text, a total of 17 were selected for analysis (Figure 1). The years in which these studies were published ranged from 2001 to 2019, with nine (52.9%) published in the recent 5 years (Figure 2).

In Table 2, we list the characteristics of these studies, including the selection of animals and models, the measurement and methods of administration, and the results. In the AD animal research quality evaluation form (Table 3), the median score of all included studies is six, with the highest and lowest scores being five and eight, respectively.

In the selected studies, there were two kinds of animals, rats (3 studies) and mice (14 studies). The number of animals used in all included articles ranged from 6 to 15 animals per group, but no article provided the method for determining animal sample size. Among them, eight studies used male animals, three studies used female animals, and five studies used both male and female. One article did not describe the gender of animal. A total of nine AD models were used in these studies. Among them, the most used animal models were APP/PS1 transgenic mice and intraventricular injection of Aβ. The routes of administration in these studies were: gavage (ig), oral (po), and subcutaneous (sc). Nine articles selected two or more doses in different groups.

### 3.2. FA Treatment Alleviates AD-Related Behaviors

Establishing a conditioned reflex is the method most commonly used to test an animal’s spatial learning and memory. Morris water maze (MWM) and the Y-maze are two widely used maze experiments that examine the establishment of conditioned reflex. This review extracted data from these two experiments in the included literature and used Review Manager 5.3 software to generate quantitative data for meta-analysis.

The MWM experiment is an experiment that forces experimental animals (rats, mice) to swim and find platforms hidden below the water surface [28]. It is widely used to monitor the learning and memory of experimental animals in various studies including AD. In MWM, the time required to find the hidden platform and the percentage of time spent in the platform quadrant are two indicators to judge the animals’ spatial learning and memory. Of the 17 studies included, 7 adopted MWM; data were retrieved from these studies for meta-analysis. Two of the studies used rat models, and five used mice models. To reduce the heterogeneity between groups, we divided the data into three subgroups, namely, “non-transgenic mice”, “transgenic mice”, and “rats” for analysis according to different animal models.

**Figure 3:**
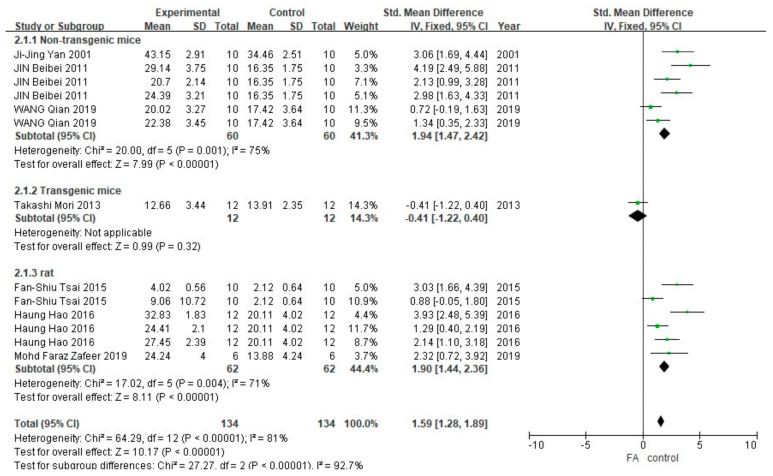
Forest plot for comparison: FA versus vehicle treatment. Outcome: MWM, time in platform quadrant.

As shown in Figure 3, a total of 13 different groups (268 animals) in 7 studies reported the data of the time spent on the platform. Meta-analysis showed that, in the non-transgenic mice and rat subgroups, the FA-treated group spent more time in the platform quadrant than the control group (*p* < 0.00001), and the heterogeneity within the group was moderate (I^2^ = 75%, *p* = 0.001 and I^2^ = 71%, *p* = 0.004). The only set of data in the transgenic mice subgroup was analyzed by the method of one-way ANOVA used in the original publication. The results showed a difference in the time spent on the platform quadrant between the FA-treated group and the control group (*p* < 0.005). Results of combining the data showed that the difference between the FA-treated group and the control group was statistically significant (*p* <0.005), but the heterogeneity between the subgroups was greater (I^2^ = 92.7%, *p* < 0.00001) than that of overall (I^2^ = 81%, *p* < 0.00001).

**Figure 4:**
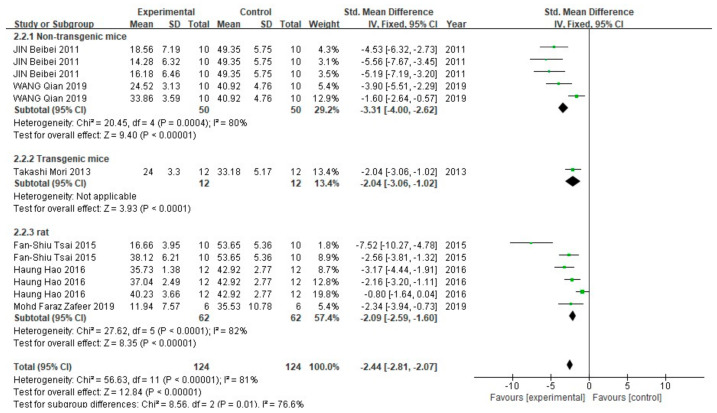
Forest plot for comparison: FA versus vehicle treatment. Outcome: MWM, escape latency.

In the escape time, 6 studies containing 12 groups (248 animals) were included in the meta-analysis (Figure 4). In all three subgroups, the fixed effect model showed that FA treatment significantly reduced the time required for animals to escape from the platform (*p* < 0.00001). The heterogeneity in the non-transgenic mice subgroup and rat subgroup was 80% (*p* = 0.004) and 82% (*p* < 0.0001), respectively.

**Figure 5:**
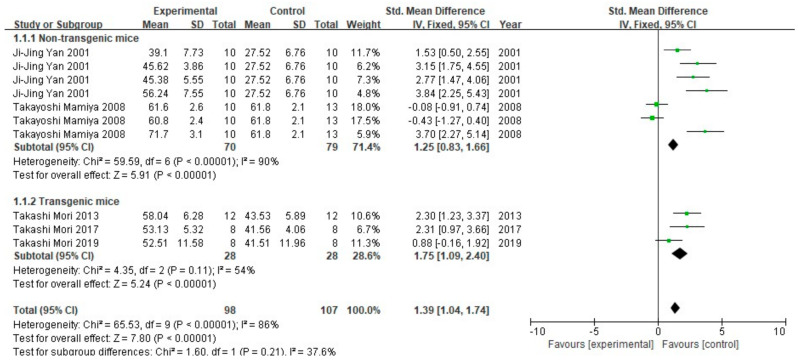
Forest plot for comparison: FA versus vehicle treatment. Outcome: Y-maze test, spontaneous alternation behavior (%).

The Y maze spontaneous alternation test is a test designed to assess different aspects of cognition based on rodents’ natural inclination to explore new environments [29]. In the experiment, spontaneous alternation behavior between arms of the maze, and number of arm entries are recorded. Five (10 groups, 205 animals) of the seventeen studies conducted the experiment. All of these studies used mice as experimental animals, and the mice were divided into non-transgenic and transgenic subgroups. The meta-analysis of results showed that the spontaneous alternation behavior between the FA-treated groups and the control groups was statistically different (*p* < 0.00001). Non-transgenic mice subgroups showed high heterogeneity (I^2^ = 90%, *p* < 0.00001), while the transgenic mice subgroups showed medium heterogeneity (I^2^ = 54%, *p* = 0.11). The results of the inter-group test of the two subgroups after treatment with the fixed effect model showed low heterogeneity (I^2^ = 37.6%, *p* = 0.21), indicating that transgenic and non-transgenic mice are comparable in terms of in spontaneous alternation behavior. Processing by the fixed effect model revealed that the heterogeneity between the two subgroups was low (I^2^ = 37.6%, *p* = 0.21), further confirming that the spontaneous alternation behavior in transgenic and non-transgenic mice in the selected studies was comparable (Figure 5).

**Figure 6:**
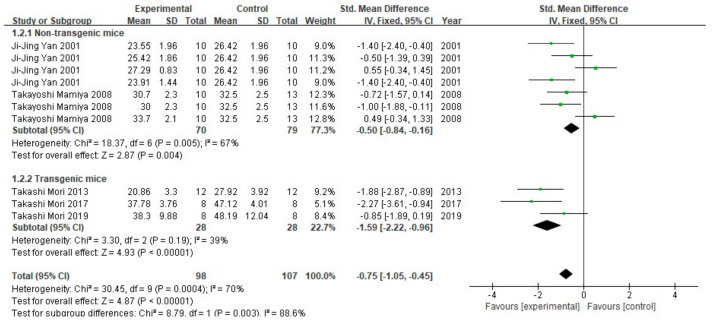
Forest plot for comparison: FA versus vehicle treatment. Outcome: Y-maze test, number of arm entries.

The analysis results of number of arm entries are shown in Figure 6. Similar to spontaneous alternation behavior, the difference in number of arm entries between the FA-treated group and the control group was statistically different (*p* < 0.005). The non-transgenic mice subgroup was moderately heterogeneous (I^2^ = 67%, *p* = 0.005), and the transgenic mice subgroup was low in heterogeneity (I^2^ = 39%, *p* = 0.19). There is greater heterogeneity between the two subgroups (I^2^ = 88.6%, *p* = 0.003) than within the subgroups, indicating that, in the included studies, the FA-treated mice explored more arms of the maze; this was true for both transgenic and non-transgenic AD model mice.

It is worth noting that the two studies included in the non-transgenic AD model tested multiple FA doses. In the study of Takayoshi Mamiya 2008 [14], spontaneous alternation behavior and number of arm entries in the low-dose group both got the opposite results as that in the high-dose group. Meanwhile, in the study of Ji-Jing Yan 2001 [11], the result of the low-dose group in the number of arm entries was also negative.

### 3.3. FA Treatment Improves Neuropathological Features in AD Animal Models

Aβ deposition is the most important pathological feature of AD. In order to evaluate the Aβ pathology in the brains of model animals more comprehensively, we selected the results with immunohistochemical (IHC) staining for Aβ plaque burden quantification and ELISA for Aβ_1-40_ and Aβ_1-42_ quantification.

**Figure 7:**
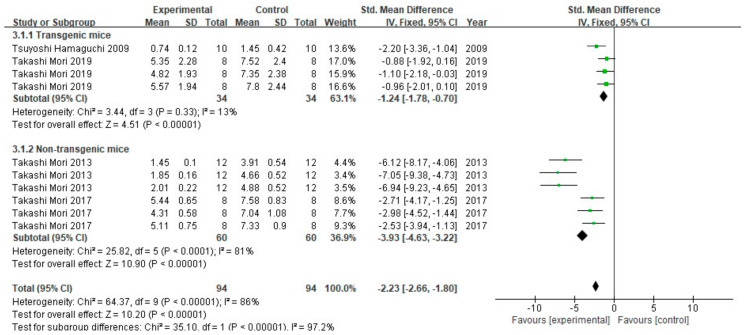
Forest plot for comparison: FA versus vehicle treatment. Outcome: Aβ burden.

A total of four studies (10 groups) tested Aβ burden using the IHC method in a total of 188 animals (Figure 7). Comparing the results of two studies that used non-transgenic mice models, FA treatment promoted the reduction in Aβ burden (*p* < 0.00001) in both, with low heterogeneity (I^2^ = 13%, *p* = 0.33). FA can also improve the Aβ burden in the brains of non-transgenic mice, but with greater heterogeneity between studies (I^2^ = 81%, *p* < 0.00001). Comprehensive analysis of the two subgroups showed that the test for overall effect had significant statistical differences (*p* < 0.00001), but its heterogeneity was greater (I^2^ = 88%, *p* < 0.00001) than that of the subgroups when analyzed separately. In addition, there was great heterogeneity between the two subgroups (I^2^ = 97.2%, *p* < 0.00001).

**Figure 8:**
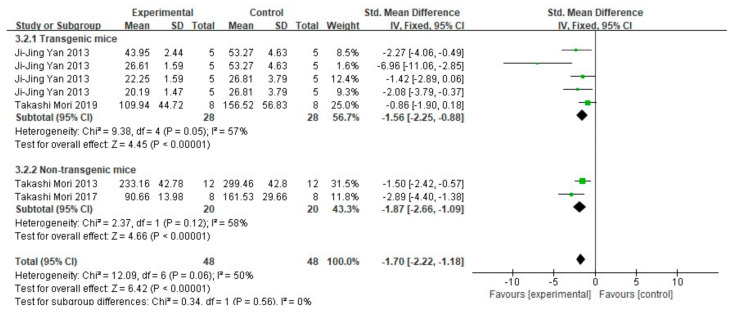
Forest plot for comparison: FA versus vehicle treatment. Outcome: Aβ_1-40._

**Figure 9:**
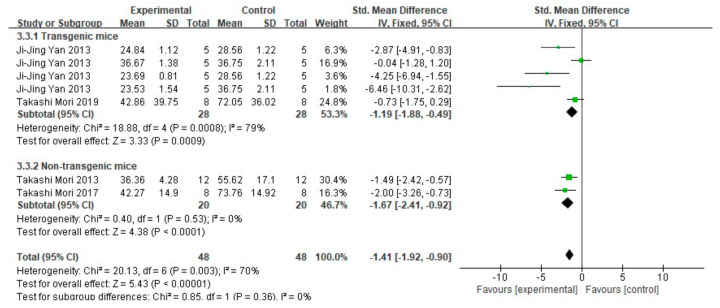
Forest plot for comparison: FA versus vehicle treatment. Outcome: Aβ_1-42_.

When extracting data of Aβ_1-40_ and Aβ_1-42_, only data that clearly described the tissue is dissolved in TBS in the article were included. Among 17 studies, 4 studies (representing a total of 96 mice in 7 groups) detected both Aβ_1-40_ and Aβ_1-42_ by ELISA. The results are shown in Figure 8 and Figure 9. The meta-analysis results of each subgroup showed that FA treatment can significantly reduce the deposition of soluble Aβ_1-40_ and Aβ_1-42_ in the brains of mice (*p* < 0.001). In terms of heterogeneity, for Aβ_1-40_, the test results in both subgroups were moderately heterogeneous (transgenic mice subgroup: I^2^ = 57%, *p* = 0.05; non-transgenic mice subgroup: I^2^ = 58%, *p* = 0.12). There was no heterogeneity between the two groups (I^2^ = 0%, *p* = 0.56). For Aβ_1-42_, and there was greater heterogeneity in the subgroup of transgenic mice (I^2^ = 79%, *p* = 0.0008), while no heterogeneity in the subgroup of non-transgenic mice subgroup (I^2^ = 0%, *p* = 0.53); similarly, there is no heterogeneity between the two groups (I^2^ = 0%, *p* = 0.85).

### 3.4. Mechanism of FA in Anti-AD

**Figure 10:**
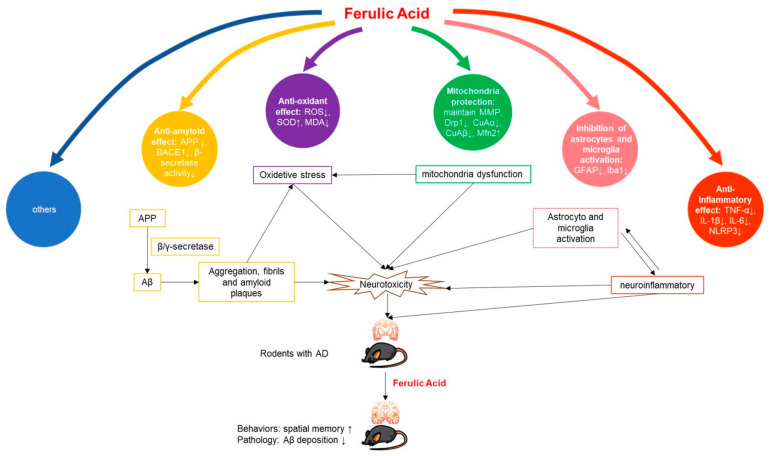
Potential mechanisms of FA in AD animal model.

With regard to the possible mechanism of FA in AD treatment, the included articles proposed at least five mechanisms (Figure 10): reducing amyloid [17,18,22,26,27], reducing inflammation [17,20,22,26], antioxidant activity [14,18,19,22,23,26], repairing mitochondrial damage [25,27], and inhibiting microglial [12,16,24] or astrocyte activation [11,13]. The significance of these mechanisms and their pathways in the treatment of AD are further analyzed in the discussion below.

## 4. Discussion

Animal experiments are indispensable for human health science research. In recent years, with the rapid development of biotechnology, the requirements for animal welfare and animal ethics have gradually strengthened. The 3R principle (replacement, reduction, refinement) is one of the representative products. Using systematic reviews to conduct targeted statistical analysis of published animal-related experiments can help researchers make full use of existing data, avoid unnecessary repetitive experiments, optimize existing experimental designs, and reduce cost. Therefore, systematic review is an effective method in line with the 3R principles. This review is the first screening and systematic data analysis of FA in AD rodent model studies. A total of 17 articles were found that met the search requirements.

### 4.1. Article Characteristics

After statistics and scoring of the characteristics of the 17 articles included in this article, we found that the average animal research quality score of the articles was only 60%, indicating that most of the articles lack strict design of animal experiments. The main reason for low scores is that none of the articles explained how to calculate the sample size, nor did they explain the general state of the animals. In addition, almost all studies did not carry out double-blind experiments, and some studies did not randomly group animals or set different doses. These problems may affect the reliability of experimental results.

For example, in the process of data extraction, we found that, in the studies with multiple doses or studies using chemically modified FA, the low-dose group or the FA monomer-treated group often showed negative results [15]. In contrast, the single-dose experiments showed significant positive results despite using lower concentrations of FA. Since almost all the included studies did not carry out double-blinding in their experiments, we believe that some results may be biased due to the subjective wishes. Therefore, we recommend that researchers design multiple drug treatment groups from low to high dose with double-blind design to reduce bias.

Of the 17 articles we reviewed, more than half were published in the last five years. This, however, does not necessarily mean that scientists have gradually increased their interest in FA. From the content of the article, we find that, with increasing years, these articles gradually tend to study the possibility of chemical modification of FA or FA combined with other compounds for AD treatment, indicating that chemical modification of natural compound monomers and synergistic effects between monomers are the current research trends and hotspots.

### 4.2. Anti-AD Potential of FA

As mentioned earlier, the clinical symptoms of AD are mainly progressive cognitive dysfunction and memory impairment, while the characteristic pathological changes are the formation of neuronal fibrillary tangles and senile plaques in the brain. Therefore, in this review, we chose two representative experiments to assess spatial memory in animals (MWM and Y-maze), and we assessed Aβ deposition, which is the most direct pathological manifestation of AD, as indicators to evaluate the effect of FA on AD.

The results of the meta-analysis showed that, compared with vehicle, FA treatment could improve the memory impairment and decrease Aβ deposition in the brains of AD animal models. It is worth noting that, although most studies have shown that Aβ deposition is related to animal behavioral performance, some studies have shown that there is no good correlation between Aβ deposition in the brain and behavioral disorders in transgenic mice expressing APP mutations [30]. Other studies have suggested that oligomeric Aβ may be the substance that causes AD neurotoxicity [31,32]. Therefore, we suggest that, when studying the therapeutic effect of drugs on AD models, researchers should choose multiple Aβ-related detection assays, especially the oligomeric Aβ detection assay.

In addition, although we divided the data into different subgroups for analysis based on the species of the experimental animals and whether they were genetically modified, the heterogeneity between the experiments was still large. These differences may be attributed to the different strains of mice used in the experiments. Although the MWM is considered to be superior to several other existing methods for testing learning and memory abilities in rodent models in terms of stability of results and duration of experiments, studies have shown that mice of different strains perform differently. For example, the performance of male C57BL/6 mice is significantly better than that of male Kunming mice, and BALB/c mice are not suitable for the experiments due to fear and poor physical performance [33,34]. Thus, choosing the right animal strain is one of the prerequisites for successful experiments when conducting such research.

### 4.3. Possible Mechanisms of FA in the Treatment of AD

The pathogenesis of AD is complex, and the etiology has not been fully elucidated. The current hypothesis involves many aspects such as Aβ toxicity, tau hyperphosphorylation, neuroinflammation, oxidative stress, and abnormal immune function. In the 17 papers included in this review, the therapeutic mechanism of FA treatment on AD animal models was mainly discussed from the aspects of anti-amyloid effect, anti-inflammation and anti-oxidant activity, repairment of mitochondrial damage, and inhibition of glial cell activation (Table 4).

#### 4.3.1. Anti-Amyloid Effect

The deposition of amyloid plaques, especially Aβ, is the most prominent pathological feature in the development of AD. *In vitro* aggregation tests have shown that FA can inhibit the formation and extension of Aβ by affecting the fiber elongation process [35]. This effect has been verified on the genetically modified *Caenorhabditis elegans* model [36].

In the body, Aβ is produced by β-secretase and γ-secretase through continuous hydrolysis of amyloid precursor protein (APP). Among them, β-secretase is considered to be the rate-limiting enzyme for Aβ production [37]. Research by Ji-jing Yan et al. [17] showed that FA inhibited Aβ deposition in the cortex, but beneficial effects were observed only in the low-dose group (5.3 mg/kg/d). Beta-site APP cleaving enzyme 1 (BACE1) is a major β-secretase. Takashi Mori et al. [18,22,26] reported that FA can reduce amyloid APP metabolism by reducing BACE1 expression and β-secretase activity in mice, thereby improving Aβ_1-40_ and Aβ_1-42_ deposition in the brain parenchyma and cerebral vessels of AD mice and slowing related toxic reactions. The team confirmed this result with *in vitro* experiments. FA not only breaks down pre-formed fibrous tangles, but also inhibits the formation and extension of Aβ in a dose-dependent manner [26]. Wang Qian et al. [27] found that FA can effectively reduce the expression levels of APP, BACE1, total Tau, and Tau pS396 in the brains of AD model mice, and increase the expression of PSD95 and PD-HE1α. The above results indicate that FA may have a therapeutic effect on AD by directly inhibiting the formation of amyloid plaques.

#### 4.3.2. Anti-Inflammatory Effect

In recent years, more and more studies have revealed that inflammation plays an important role in the occurrence and development of AD. The anti-inflammatory effects of FA have been reported. Takashi Mori et al. [18,22,26] have shown in multiple studies that FA alleviated neuroinflammation in PS1/APP mice, including Aβ plaque-related proinflammatory cytokines TNF-α and IL- 1β expression. Ji-jing Yan et al. [17] also found that FA reduced the level of IL-1β in mouse brain cortex. Ming Rui et al. [24] found that FA can reduce the increased expression of IL-1β, IL-6 and TNF-α in the brain tissue of AD model mice. Huang Hao et al. [20] found that FA treatment can alleviate the LPS-induced increase in IL-1β, caspase-1, NLRP3, and PDE4B mRNA levels, which means that FA has the potential to block the activity of NLRP3 inflammasome.

#### 4.3.3. Antioxidant Effect

**Figure 11:**
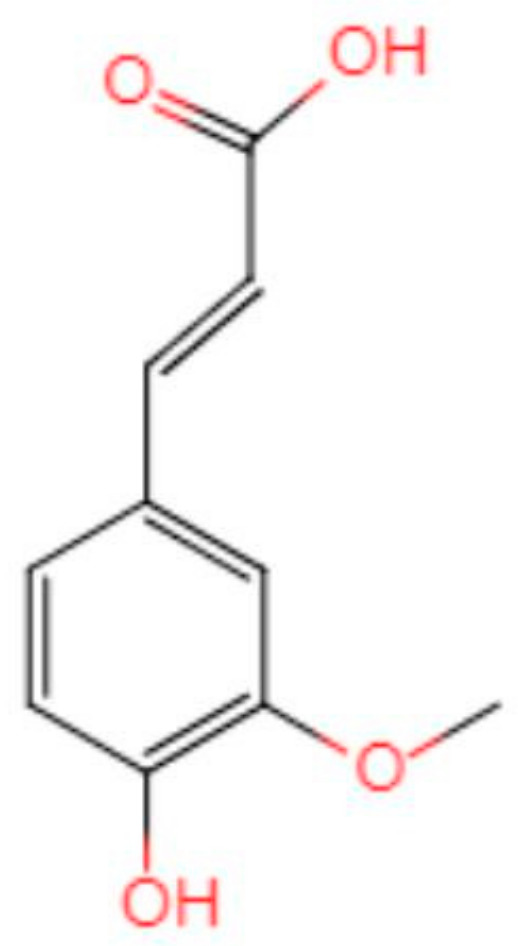
Chemical structure of ferulic acid.

The hypothesis that oxidative stress is involved in AD development has been confirmed by multiple laboratories *in vitro* and *in vivo* [38,39,40]. A review by Butterfield [41] shows that numerous studies have found that Aβ produces neurotoxicity by inducing oxidative stress (OS) in the brain. The chemical structure of FA determines its antioxidant function (Figure 11). The presence of 3-methoxy and 4-hydroxy groups on the benzene ring can easily form resonance-stable phenoxy groups. A carboxylic acid group having an adjacent unsaturated C-C double bond can stabilize the free radical by preventing the free radical film attack. At the same time, the carboxylic acid group can act as a lipid anchor, providing protection against lipid peroxidation [42]. An RCT study showed that FA supplementation can improve lipid metabolism in patients with hyperlipidemia, and improve oxidative stress and inflammation [43]. These results suggest that FA may help to improve the oxidative stress state and inflammation in the brain of AD patients.

ROS is a common byproduct of electron leakage from the mitochondrial inner membrane during mitochondrial oxidative phosphorylation. Under normal conditions, ROS is rapidly eliminated by enzymes, but when mitochondria are disturbed, ROS production may exceed the cell’s ability to neutralize it, resulting in oxidative damage [44]. Mohd Faraz Zafeer et al. [25] found that long-term oral administration of FA can significantly alleviate the exacerbation of ROS in AD mice models.

Mamiya et al. [14] found that the increased expression of carbonyl protein, one of the markers of protein oxidative damage, can be inhibited by FA pretreatment in AD animal models, indicating that FA can inhibit OS in the brain of AD animal models. Huang Hao [20] found that SOD expression in the brain of FA-treated AD mice increased. Takashi Mori et al. [18,22] showed that mRNA and protein expression of three oxidative stress markers (SOD1, catalase, and GPX1) were reduced in FA-treated PSAPP mice. Fan-Shiu Tsai et al. [19] found that administration of FA to rats increased SOD activity; attenuated Aβ_1-40_-induced activity of Mn-SOD and Cu, as well as Zn-SOD in the cortex and hippocampus; and improved the inhibition of GSH activity in the cortex. Wang Yue et al. [23] found that, after treatment with FA, AD model mice had increased SOD activity and reduced MDA content, suggesting that FA can reduce ROS in the brain of AD mice.

#### 4.3.4. Mitochondria Protection

Autopsy has revealed abnormal hippocampal mitochondria in patients with AD [45]. In the cell, mitochondria function in a steady state of division-fusion to meet the energy needs of the body [46]. Drp-1 is a key effector of mitochondrial fission localized at the outer mitochondrial membrane, and Mfn2 is a marker of mitochondrial fusion. The dynamic balance between the two proteins is essential for maintaining the mitochondrial health of neurons [47].

Mohd Faraz Zafeer et al. [25] found that FA treatment can restore the balance between mitochondrial division and fusion by regulating the level of PGC-1alpha protein, thereby preventing the loss of mitochondrial membrane potential and reducing Drp-1-dependent mitochondrial fission. Wang Qian et al. [27] found that the expression of Drp1, CnAα, and CnAβ mRNA and protein in the cerebral cortex of AD mice were significantly reduced in the FA-treated group, and the expression of MFN2 protein was increased. The data suggested that FA can reverse the Aβ-induced, abnormally increased expression of Drp1 and its phosphorylation regulatory pathway genes CnAα and CnAβ. In addition, FA may enhance the resistance of neurons to Aβ toxicity by locating at mitochondria where they can repair mitochondrial biological transport balance, thereby maintaining neuronal signaling delivery and improving learning and cognition in AD mice.

#### 4.3.5. Inhibition of Astrocytes and Microglia Activation

Astrocytes are the most widely distributed and the largest type of glial cells in the brain; they have many functions such as support and protection neuron function, promote neuron repair, and improve neurotransmitter regulation. Glial fibrillary acidic protein (GFAP) is a marker of astrocyte activation. When neurons are damaged, astrocytes are activated to produce a large amount of GFAP. Continuous injection of rats with Aβ_1-40_ increases GFAP immunoreactivity [48]. Mohd Faraz Zafeer et al. [25] found that long-term oral FA treatment can reduce the expression of GFAP. Jing Beibei et al. [16] found that, compared with the vehicle group, the level of GFAP decreased with the increase in FA concentration. Morphologically, Ming Rui et al. [24] observed that, compared with the sham operation group, the number of GFAP-positive astrocytes in the cortex of the model group was significantly increased. After FA treatment, the amount of GFAP positive astrocytes and fluorescence intensity gradually decreased, the axons and dendritic structures of surrounding neurons were basically normal, and the connections between neurons increased significantly. Ji-Jing Yan et al. [11] confirmed that inhibition of astrocytes by long-term treatment with FA was not mediated by affecting the secretion of neurotrophic factors such as NGF, BDNF, and bFGF. Jae-Young Cho et al. [13] further found that eNOS, 3-NT, and GFAP colocalized in the brain of AD mice model, and that the change pattern was similar to that of GFAP, suggesting that the ability of FA to protect the brain from Aβ-induced toxicity may be mediated by inhibiting the expression of eNOS and 3-NT in astrocytes. Takashi Mori [18,22,26] demonstrated that FA treatment can reduce GFAP and Iba1 levels, and inhibit plaque-associated proliferation of microglia and astrocytes. Hee-Sung KIM et al. [12] suggested that FA induced inhibition of astrocyte activation may be achieved by inhibiting microglial activation. However, the researcher did not provide sufficient evidence to prove this hypothesis.

It is worth noting that Ji-Jing Yan et al. [11] found that, on the fourteenth day, FA-treated animals showed transient activation of astrocytes manifested by increased expression of GFAP. At the same time, experimental animals performed poorly in behavior with elevated IL-1β. Although the morphology of astrocytes in the hippocampus of mice seemed to return to normal thereafter, they continued to show a weak inhibitory response to Aβ stimulation. This may be the resistance of astrocytes to Aβ toxicity caused by FA treatment. If this is indeed the mechanism by which FA inhibits astrocyte activation, then researchers should further consider whether this resistance can persist for a long time. These data will be helpful to determine frequency and duration of FA medications for AD patients in the future.

#### 4.3.6. Others

Fan-Shiu Tsai et al. [19] suggested that the mechanism by which FA inhibits Aβ_1-40_- induced behavioral injury in rats may be related to the inhibition of AChE activity. Wang Yue et al. [23] found that the phosphorylated apoptosis-related proteins were significantly increased in the brain of APP/PS1 transgenic mice, while those proteins were significantly reduced in the FA treatment group, indicating that FA may protect neurons by reducing the phosphorylation of apoptosis-related proteins in the brain of AD mice. Mohd Faraz Zafeer et al. [25] found that long-term oral administration of FA can reduce caspase 3 activation. Huang Hao et al. [20] suggest that FA may have a significant regulatory effect on the PDE4/cAMP/CREB signaling pathway.

### 4.4. Development Perspective

#### 4.4.1. Mechanism Exploration

FA has been proven to be effective in treating AD to a certain extent, from cell to animal models; however, the mechanism has not been fully elucidated. Understanding the pharmacological mechanism will greatly facilitate drug development and minimize biological safety concerns for the clinical application of FA. Therefore, there is an urgent need for determining the molecular mechanism of FA for AD treatment.

#### 4.4.2. Structural Modification

Compared with other, larger phenolic compounds, FA has better cell permeability due to its lower molecular weight (194.18 g/mol). Studies have shown that FA has good bioavailability in rats. FA can be quickly absorbed through the gastric mucosa when orally administered, then transported to the portal vein of the liver, combined with glucuronic acid and/or sulfate, and can be maintained in the body for a long time [49]. However, FA may have difficulty penetrating the blood–brain barrier due to its hydroxyl group. Therefore, structural modification of FA to generate candidate drugs with more potent neuroprotective activity and higher blood–brain barrier penetration is an important direction for future research.

#### 4.4.3. Extrapolation to Humans

In the process of drug development, the ultimate goal of animal experiments is to make the drugs better used in humans. When analyzing the data extracted from the included articles, we found that there is a large heterogeneity between the reported data sets. In addition to laboratory conditions and the genetic background of the animal, the heterogeneity may also be partially derived from differences in administration of the FA. These heterogeneities between experiments can be computer-simulated to establish a physiologically based pharmacokinetic/pharmacodynamic (PBPK/PD) model of drug-to-disease and can use computational pharmacology to improve data integration between different studies for FA on AD animal models. In addition, the establishment of a PBPK/PD model of FA to AD is also conducive to the extrapolation of animal experiments to human experiments, and can help researchers find better routes and dose.

On the other hand, it is worth noting that many natural compounds have been proved to have hormetic dose–response, that is, the compounds have two-way effects on anti-oxidation and anti-neurodegenerative diseases at very low doses and higher doses [50]. Therefore, it is necessary to carefully select the targeted concentration when extrapolating to the patient. Based on this, more detailed pharmacokinetic studies in animals and humans are needed to determine the effective plasma drug concentration to obtain better and more stable drug effects.

In summary, the current researches of FA on AD are still at the basic research stage. There is no clinic trail being registered on the clinicaltrials.gov (accessed on 28 July 2021) website. It is a long way from preclinical studies on animals to clinical trials on humans, and ultimately, to market. Nevertheless, the results of this review, showing that FA may be effective in repairing the memory and pathological damage caused by AD, suggest that the way is worth travelling.

## 5. Conclusions

In this systematic review, we adopted systematic collection and meta-analysis methods to comprehensively examine 17 studies investigating the effect of FA on animal AD models. The evaluation results indicate that FA is a promising drug candidate in the treatment of AD, in that it appears able to improve the cognitive impairment and alleviate the neuropathological features in the brain of AD animal models. We summarized and discussed the possible pharmacological mechanisms involved in this activity, as presented in the articles. We also pointed out the limitations of current FA preclinical studies, namely, the poor quality of methodology, the high degree of bias, and inadequate exploration of mechanisms. This information will provide guidance and reference for the design of further experiments as well as, in due course, clinical trials.

## Figures and Tables

**Table 1:** Inclusion and exclusion criteria.

Inclusion Criteria	Exclusion Criteria
Rodent with a clear genetic origin	Cell model or non-rodent
Included a ferulic acid group and a control group administered by any route, and each group was independent of the other group.	Groups without ferulic acid treatment or no control group
AD model or contains AD model	Not AD model
Study that assessed AD-related results, such as behavioral changes and protein changes.	Study that did not assess AD-related results
Full access to published study	Unable to access full text, review, case report, editorial, abstract, letter, and/or comments

**Table 2:** Characteristics of included publications.

Study	Animal Models and Species	Quantity Sex Age	Administration	Outcome		
				Behavioral Change	Neuropathological Change	Biochemical Change
Ji-Jing Yan 2001 [11]	i.c.v. injection of Aβ_1-42_ ICR mice	10 M 18~26 g	0.002%, 0.004%, and 0.006% (*w*/*v*) Free drinking 1, 2, 3, 4 w	Improved memory (passive avoidance task; Y-maze tests; MWM)	Hippocampus GFAP and IL-1β immunoreactivities↑ (Immunocytochemistry)	Cortex Acetylcholine level↓ (colorimetry);
Hee-Sung KIM 2004 [12]	i.c.v. injection of Aβ_1-42_ ICR mice	6 M 18~26 g	0.006% (*w*/*v*) Free drinking 4 w	N/A?	Reduced microglial activation (Immunocytochemistry: OX-42 immunoreactivity↓)	Inhibition IFN-γ Immunoreactivity (Immunocytochemistry);
Jae-Young Cho 2005 [13]	i.c.v. injection of Aβ_1-42_ ICR mice	6 M 18~26 g	0.006% (*w*/*v*) Free drinking 4 w	N/A?	Reduced astrocytes activation	Alleviated oxidative stress in the hippocampus (eNOS and 3- NT immunoreactivity↓)
Takayoshi Mamiya 2008 [14]	i.c.v. injection of BSO ICR mice	10/15 M 25 w	0.5, 1, or 5 mg/kg sc 6 d	Improve recognition memory (the novel object recognition test); improve short-term memory (Y-maze);	extent of protein oxidation↓; carbonyl protein levels↓ in forebrains;	N/A?
Tsuyoshi Hamaguchi 2009 [15]	Mice double mutation K670N-M671L Tg2576 mice	10 F 5 mon	0.5% in food 10 mon	N/A?	Aβ deposits↓ (IHC)	N/A?
JIN Beibei 2011 [16]	Injected KA into hippocampus CA1 region KM mice	10 M&F 20~30 g	20, 40 and 80 mg/kg ig 30 d	Improved learning and cognitive skills (MWM)	Reduced expressions of GFAP in hippocampal CA1 region (Immunohistochemistry)	N/A?
Ji-Jing Yan 2013 [17]	APP/PS1 mice	5 F 6 mon	5.3 and 16 mg/kg/d Free drinking 6 mon	Improved memory (novel-object recognition test, Y-maze task)	Aβ_1-42_ and Aβ_1-40_ levels↓ (immunoassay kits)	Il-1β↓ (immunoassay kits)
Takashi Mori 2013 [18]	PSAPP C57BL/6J mice	12 M&F 6 mon	30 mg/kg ig 6 mon	Remediation of behavioral impairment (field activity testing; object recognition test; Y-maze test; MWM)	Cerebral Aβ deposits↓ (4G8 immunohistochemistry, ELISA)	Reduced neuroinflammation and Oxidative Stress: Iba1↑ (Immunohistochemistry); TNF-a, IL-1β, Sod1, catalase, and Gpx1 mRNA↓ (QRT-PCR)↓; reduced microglial and astroglial activation:GFAP↓ (Immunohistochemistry)
Fan-Shiu Tsai 2015 [19]	i.c.v. injection of Aβ_1-42_ SD rats	10~12 M 250~300 g	50 and 100 mg/kg ig 2 w	Attenuated impairment of cognitive function (Inhibitory Avoidance Test); improve memory (MWM);	N/A?	Cortical and hippocampal GSH↑, SOD↑, Cu, Zn-SOD↓ activity (spectrophotometrically); brain AChE Activity↓ (Ellman method)
Huang Hao 2016 [20]	LPS-induced SD rats	12 F 280~320 g	25, 50, 100 mg/kg ig 34 d	Improved learning and cognitive skills (MWM)	Protective effect on brain histopathology (HE staining, β-tubulin), PDE4B	Anti-oxidize effect (SOD↑); suppressed mRNA elevation of PDE4B, NLRP3, IL-1β and caspase-1(Q-PCR); PDE4B↓ (Immunohistochemistry, WB); NLRP3↓, CREB↑ and pCREB↑ (WB)
Masaki Kikugawa 2016 [21]	i.c.v. injection of Aβ_25~35_ C57BL/6 J mice	6 M 16–19 g	0.1 μmol/g/day po 42 d	Improved contextual freezing response impairment (fear conditioning test)	Protective effects on neurons survival (Nissl stain)	N/A?
Takashi Mori 2017 [22]	APP/PS1 C57BL/6J mice	8 M&F 12 mon	30 mg/kg ig 3 mon	Improved memory (assess novel object recognition memory, the novel object recognition test and retention test phases; Y-maze test, RAWM)	Cerebral parenchymal A β deposits↓and size↓ (IHC), A β _1-40_, A β _1-42_↓ (ELISA); vascular A β deposits↓ (IHC); attenuated astrocytosis and microgliosis (IHC of GFAP and Iba1); Attenuated Synaptotoxicity: synaptophysin immunoreactivity↑ (IHC)	Promoted nonamyloidogenic and inhibited amyloidogenic APP processing: sAPP-α/holo-APP↓ (WB), β-oligomers↓ (ELISA); activated ADAM10 and inhibits BACE1(WB); attenuated neuroinflammation and oxidative stress: TNF-α↓, IL-1β↓, SOD1↓, GPx1↓ (Q-PCR); attenuated Synaptotoxicity: synaptophysin immunoreactivity↑ (IHC)
Wang Yue 2017 [23]	APP/PS1 C57BL/6 mice	10 15~20 g	20, 40, 100 mg/kg ig 7 d	N/A?	N/A?	Reduced apoptosis (WB: Bcl-2↑, Bax↓, p-JNK↓, p-C-Jun↓, Caspase3↓), Reduces oxidative stress in the brain (MDA↓, SOD↑)
MING Rui 2018 [24]	Injected KA into hippocampus CA1 region KM mice	M&F 26 ± 4 g	20, 40, and 80 mg/kg 30 d	N/A?	Reduced number of positive GFAP cells in cerebral cortical glial cells (Immunofluorescence)	Reduced inflammatory cytokines (ELISA: IL-1β↓, IL-6↓, TNF-α↓)
Mohd Faraz Zafeer 2019 [25]	ICV-STZ Wistar rats	6 M 350 ± 25 g	100 mg/kg po 21 d	Attenuated spatial memory and learning loss (MWM)	Protective effect on brain histopathology (HE staining of coronal sections)	Mitigation of AD-related oxidative stress (DCFDA: ROS↓); mito-protective efficacy (flow cytometric: Δψm; Calcein-AM/CoCl2 assay: mPTP; WB: Drp-1↑, Mfn2↓, PGC1-α↑, BAX↓, Cytochrome-C↓, LPO↓); DNA fragmentation↓ (comet assay)
Takashi Mori 2019 [26]	APP/PS1 mice	8 M&F 12 mon	30 mg/kg ig 3 mon	Improved memory (Y-maze, RAWM; novel object recognition test; alternation Y-maze task)	Cerebral Aβ deposits↓ (4G8 immunostain); Aβ_1-40_ and Aβ_1-42_ levels↓ (ELISA)	Promoted nonamyloidogenic and inhibited amyloidogenic APP cleavage (WB); ADAM10 ↓, BACE1 ↓ (WB); mitigated astrocytosis and microgliosis (IHC of GFAP and Iba1); dampened neuroinflammation and oxidative stress: TNF-α↓, IL-1β↓ (Q-PCR), SOD1↓, GPx1↓ (Q-PCR and WB); attenuated Synaptotoxicity: synaptophysin immunoreactivity↑ (IHC)
WANG Qian 2019 [27]	Injecting Aβ_1-42_ into the lateral ventricle KM mice	10 M 18~22 g	0.1 and 0.4 g/kg ig	Improved spatial positioning memory (MWM). No effect on the excitability of the central nervous system (spontaneous activity experiment)	Improved morphological changes (HE Staining); Tau; pS396 protein phosphorylated, total Tau protein↓ and S396↓; reduced Aβ generation	Improved abnormal mitochondrial division (RT-PCR: Drp1↓, CnAα↓, CnAβ↓mRNA); Bace1↓

**Table 3:** AD animal research quality evaluation form.

Study	(1)	(2)	(3)	(4)	(5)	(6)	(7)	(8)	(9)	(10)	Quality Score	Quality Score (%)
Ji-Jing Yan 2001	√	×	√	×	×	×	√	√	√	√	6	60
Hee-Sung KIM 2004	√	×	×	×	×	×	√	√	√	√	5	50
Jae-Young Cho 2005	√	×	×	×	×	×	√	√	√	√	5	50
Takayoshi Mamiya 2008	√	×	√	×	×	×	√	√	√	√	6	60
Tsuyoshi Hamaguchi 2009	√	√	×	×	×	×	√	√	√	√	6	60
JIN Beibei 2011	√	×	√	×	×	×	√	√	√	√	6	60
Ji-Jing Yan 2013	√	×	√	×	×	×	√	√	√	√	6	60
Takashi Mori 2013	√	×	×	×	×	×	√	√	√	√	5	50
Fan-Shiu Tsai 2015	√	√	√	√	×	×	√	√	√	√	8	80
Haung Hao 2016	√	√	√	×	×	×	√	√	√	√	7	70
Masaki Kikugawa 2016	√	×	×	×	×	×	√	√	√	√	5	50
Takashi Mori 2017	√	×	×	×	×	×	√	√	√	√	5	50
Wang Yue 2017	√	√	√	×	×	×	√	√	√	√	7	70
MING Rui 2018	√	×	√	×	×	×	√	√	√	√	6	60
Mohd Faraz Zafeer 2019	√	×	×	×	×	×	√	√	√	√	5	50
Takashi Mori 2019	√	√	×	√	×	×	√	√	√	√	7	70
WANG Qian 2019	√	√	√	×	×	×	√	√	√	√	7	70

√ = fulfilling the criterion, × = not fulfilling the criterion. (1) peer-reviewed publication; (2) presence of randomization of subjects into treatment groups; (3) assessment of dose–response relationship; (4) blinded assessment of behavioral outcome; (5) monitoring of physiological parameters such as body temperature; (6) calculation of necessary sample size to achieve sufficient power; (7) statement of compliance with animal welfare regulations; (8) avoidance of anesthetic agents with marked intrinsic neuroprotective properties (e.g., ketamine); (9) statement of potential conflict of interests; (10) use of a suitable animal model.

**Table 4:** Possible mechanisms of FA in the treatment of AD.

Pharmacological Effects	Mechanism	Studys
Anti-amyloid effect	Inhibition of Aβ deposition	[17]
	Inhibition of the formation and extension of Aβ	[35,36]
	Inhibition of β-secretase	[18,22,26,27]
	Reduce APP and Tau expression	[27]
Anti-inflammatory effect	Reduce TNF-a, IL-6 and IL- 1β expression	[17,18,20,22,24,26]
	Block the activity of NLRP3 inflammasome	[20]
Antioxidant effect	Inhibition ROS and MDA production, increase SOD expression	[14,18,19,20,22,23,25,42]
Mitochondria protection	reverse the abnormally increased expression of Drp1	[25,27]
Inhibition of astrocytes and microglia activation	Reduce GFAP positive astrocytes	[11,16,18,22,24,25,26]
	Reduce eNOS, 3-NT in astrocytes	[13]
	Reduce Iba1 positive microglia	[12,18,22,26]
Others	Inhibition AChE activity	[19]
	Reducing the phosphorylation of apoptosis-related proteins	[23,25]
	Regulate PDE4/cAMP/CREB signaling pathway	[20]

## Data Availability

The original data used in this article are all extracted from published papers and can be obtained from the references in the article.
